# Molecular and Morphometric Update on Italian *Salicornia* (Chenopodiaceae), with a Focus on the Species *S. procumbens* s. l.

**DOI:** 10.3390/plants12020375

**Published:** 2023-01-13

**Authors:** Katia Sciuto, Marion A. Wolf, Adriano Sfriso, Lisa Brancaleoni, Mauro Iberite, Duilio Iamonico

**Affiliations:** 1 Department of Chemical, Pharmaceutical and Agricultural Sciences, University of Ferrara, Via Luigi Borsari 46, 44121 Ferrara, Italy; 2 Department of Environmental Sciences, Informatics and Statistics, Ca’Foscari University of Venice, Via Torino 155, 30172 Mestre, Italy; 3 Department of Environment and Prevention Sciences, University of Ferrara, C.so Ercole I D’Este 32, 44121 Ferrara, Italy; 4 Department of Environmental Biology, Sapienza University of Rome, P.le A. Moro 5, 00185 Roma, Italy; 5 Ce.R.S.I.Te.S., Sapienza University of Rome, Via XXIV Maggio 7, 04100 Latina, Italy

**Keywords:** biometry, endemism, ETS, *Salicornia* L., *psb*A*-trn*H

## Abstract

*Salicornia* is a highly taxonomically problematic genus due to the reduced morphological observable characters. Ten Eurasian species are currently recognized: *S. alpini*, *S. europaea*, *S. fruticosa*, *S. hispanica*, *S. lagascae*, *S. perennans*, *S. perennis*, *S. persica*, *S. procumbens*, and *S. pruinosa*. In addition, eleven subspecies are accepted, mainly based on their distribution areas. Along the Venetian coasts and in Sardinia, in the past, an endemic species called *S. veneta* was recognized, but this name was later synonymized with *S. procumbens* subsp. *procumbens*. The aim of the present research is investigating different Italian *Salicornia* populations by a molecular point of view, using the nuclear ribosomal external transcribed spacer ETS and the plastid *psb*A-*trn*H intergenic spacer. A particular focus is on the comparison between Venetian (including those occurring in *locus classicus* of *S. veneta*) and Sardinian *S. procumbens* and other Italian populations of this species. The molecular analyses based on the plastid marker highlight that the Italian *S. procumbens* populations form two well distinct groups. In particular, some of the Venetian (*Locus classicus* of *S. veneta*) and all the Sardinian specimens are genetically distinct (=plastid haplotype 1) from the other investigated populations (=plastid haplotype 2). This indicates that the *psb*A*-trn*H haplotype 1 glassworts represent a distinct entity, which we suppose to coincide with the former *S. veneta*. Therefore, we suggest to recognize this taxonomic entity at the subspecies rank, as *S. procumbens* subsp. *veneta* comb. and stat. nov. However, contrary to the results found with the plastid *psb*A*-trn*H intergenic spacer, the ETS locus does not show a separation into two distinct clades for *S. procumbens*, probably due to a different evolution of the two loci. Nevertheless, in the ETS phylogenetic reconstruction, the Sardinian specimens (=ribotypes 2 and 3) are placed, together with a Moroccan sample, in a subclade separated from all the other *S. procumbens*. These results suggest that the Sardinian populations can represent a subspecies/incipient speciation process, probably due to geographic isolation. In the light of this, morphometric analyses (k-means, MANOVA, PCA, DA, and Box-Plot) have been carried out on the Sardinian and Venetian populations to verify if this distinction is detectable also by a morphological point of view. The morphometric analyses highlight the existence of two groups, concerning both the nuclear and plastid trees. Six characters were found to be diagnostic.

## 1. Introduction

*Salicornia* L. (Salicornioideae Ulbr., Chenopodiaceae Vent.) is a genus of 25–30 annual species distributed worldwide in salt marshes and saline inland habitats [1,2], wetlands that represent important ecosystems for biodiversity conservation because of their ecological function and economic values [3]. This genus is critical from the taxonomical point of view due to the low number of morphologic characters, the high phenotypic variability, and the recurring hybridization [1,4,5,6,7].

In the Eurasian area, Kadereit et al. [7] recorded the following four *Salicornia* species: *S. europea* L. [8], including three subspecies; *S. perennans* Willd. [9], with two subspecies; *S. procumbens* Sm. [10], with four subspecies; *S. persica* Akhani [11], with two subspecies. Four further taxa, *S. sinus-persica* Akhani [12], *S. persica* subsp. *rudshurensis* Akhani [12], *S. perspolitana* Akhani [12], and *S.* x *tashkensis* Akhani [12], are recognized as taxonomically doubtful by Kadereit et al. [6]. In 2017, Piirainen et al., [13] merged the *Sarcocornia* genus with the *Salicornia* one. As a consequence, another six taxa were added to the Eurasian *Salicornia* species: *S. alpini* subsp. *carinata* (Fuente, Rufo, and Sánchez Mata) Piirainen and G. Kadereit, *S. fruticosa* (L.) L., *S. hispanica* (Fuente, Rufo, and Sánchez Mata) Piirainen and G. Kadereit, *S. lagascae* (Fuente, Rufo, and Sánchez-Mata) Piirainen and G. Kadereit, *S. perennis* Mill. and *S. pruinosa* (Fuente, Rufo, and Sánchez Mata) Piirainen and G. Kadereit.

Based on the taxonomic revisions of Italian glassworts [4,14,15], four species traditionally occur in Italy: *S. emerici* Duval-Jouve, *S. dolichostachya* Moss [16], *S. veneta* Pignatti and Lausi [16], and *S. patula* Duval-Jouve [17]. However, the most recent classification by Kadereit et al. [7] has synonymized *S. emerici*, *S. dolichostachya*, and *S. veneta* with *S. procumbens* Sm. subsp. *procumbens* and *S. patula* with *S. perennans* Willd. subsp. *perennans*.

Focusing on *Salicornia veneta*, it is an Italian endemism occurring along the coasts of the Adriatic Sea from north to south-eastern Italy (Veneto, Emilia-Romagna, Marche, and Apulia regions) and in western Sardinia, according to different Italian authors [4,14,15,18,19,20,21]. In Kadereit et al. [7], the morphology of these populations was not investigated in deep and *S. veneta* was synonymized with *S. procumbens* subsp. *procumbens* without providing any discussion. On the other hand, the authors decided to maintain as separate taxa *S. freitagii* Yaprak and Yardakulol [22] (endemic from central Anatolia), *S. pojarkovae* Semenova [23] (from White and Barent seas), and *S. heterantha* SS. Beer and Demina [24] (from Rostov Province in SE-Russia). On the basis of morphological differences and geographic distribution, these taxa were accepted as subspecies of *S. procumbens*: *S. procumbens* subsp. *freitagii* (Yaprak and Yardakulol) G. Kadereit and Piirainen [7], *S. procumbens* subsp. *pojarkovae* (Semenova) G. Kadereit and Piirainen [7], and *S. procumbens* subsp. *heterantha* (SS. Beer and Demina) G. Kadereit and Piirainen [7]. Indeed, Kadereit et al. [7] stated “*Salicornia pojarkovae* and *S. freitagii* also lack distinct molecular characteristics, but are both morphologically and geographically distinct […] and therefore are treated as subspecies of *S. procumbens*”.

Inside the *Salicornia procumbens* group, only two specimens from Italian localities were included by Kadereit et al. [7] in their systematic revision: one from Venetian lagoon (Veneto region, north-eastern Italy) and one from saltworks of Trinitapoli (Puglia region, south-eastern Italy), both deposited at KAS. Since the Sardinian populations were not included in Kadereit et al. [7] and Sardinia hosts a very high rate of plant endemics in the context of the Mediterranean basin (294 endemics out of 2301 native taxa) [25,26], we decided to investigate these populations together with that from the type locality of *S. veneta*, using both a molecular and biometric approach.

Molecular analyses were based on the nuclear external transcribed spacer ETS, indicated by Kadereit et al. [6,7] as the most informative for *Salicornia*, and the plastid *psb*A*-trn*H intergenic spacer, following a recent DNA barcoding study on coastal halotolerant Poaceae and Chenopodiaceae [27] in which the plastid *psb*A*-trn*H showed the best species discrimination rates.

The present study is part of a taxonomical and nomenclatural research on Amaranthaceae s. lat. [4,28,29,30,31,32,33,34,35,36,37].

## 2. Materials and Methods

### 2.1. Plant Material

**Figure 1:**
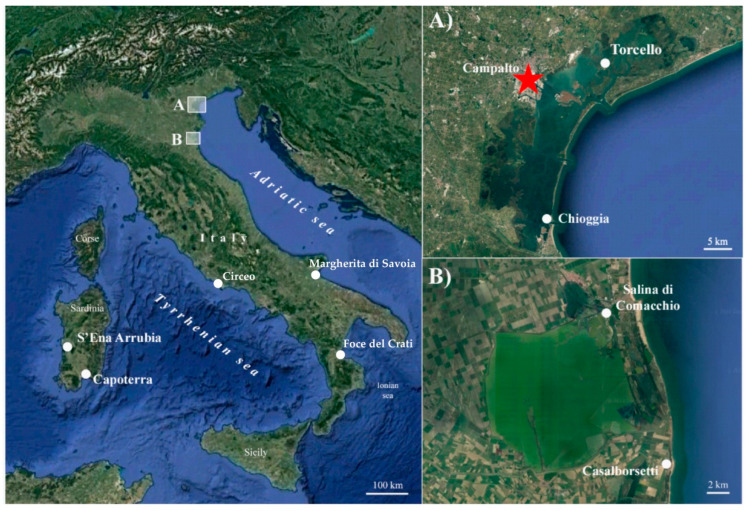
Map of the *Salicornia* populations investigated in this study. (**A**) Focus on Veneto sampling sites; the red star indicates the *locus classicus* of *S. veneta*. (**B**) Focus on Emilia-Romagna sampling sites.

Field surveys were carried out in saltmarshes of Veneto region (Venice Lagoon), Emilia-Romagna region (Salina di Comacchio and Casalborsetti localities), Lazio region (Circeo National Park near Sabaudia), Puglia region (Margherita di Savoia locality), Calabria region (Foce del Crati protected area), and Sardinia region (S’Ena Arrubia and Capoterra localities) (Figure 1 and Table 1). All the specimens collected were deposited in the Herbarium RO (acronym according to [38]). The species nomenclature follows [7,13,15].

### 2.2. Molecular and Phylogenetic Analyses

Total DNA was isolated from stem and/or inflorescences of herbarium material using ca. 20–50 mg of plant material and following the protocol of the Genomic DNA purification kit (Thermo Scientific™, Waltham, MA, USA). The nuclear ETS region was amplified following Kadereit et al. [7], while the *psb*A*-trn*H intergenic spacer was obtained according to Bruni et al. [39]. The obtained PCR products were purified using the HT ExoSAP-IT (Applied Biosystems™, Waltham, MA, USA) and sequencing was carried out at the Eurofins Genomics Sequencing Service (Ebersberg, Germany), with the same primers employed in the PCR reactions. The GeneStudio sequence analysis software (http://genestudio.com/) was used to assemble final consensus sequences. The obtained sequences were deposited in the INSDC archives, through the GenBank platform, with the accession numbers reported in Table 1.

An ETS sequence dataset was created including the newly obtained sequences and other suitable sequences available in the INSDC repositories, following the most recent classifications for the genus *Salicornia* [7,13], for a total of 72 sequences. A *psb*A*-trn*H sequence dataset was created including the sequences obtained in this study for the Italian populations, for a total of 16 sequences. Since the group composed by *Salicornia perennis* (ex. *Sarcocornia perennis*) and *Salicornia fruticosa* (ex. *Sarcocornia fruticosa*) sequences resulted as the outgroup in the ETS phylogeny, the *psb*A*-trn*H sequence of sample SV23 (corresponding to *S. fruticosa*) from the Venice lagoon was used to root the plastid spacer trees. Multiple sequence alignments were generated with MUSCLE [40] and used for phylogenetic analyses. The ETS multi-alignment consisted in 507 aligned positions, while the *psb*A*-trn*H multi-alignment had 554 aligned positions, including gaps and indels.

Phylogenetic analyses were performed with MEGA version XI program [41] using the Maximum Parsimony (MP) and the Maximum Likelihood (ML) methods. For ML, the model that best fit the data was found with the “Find best DNA Models” tool implemented in MEGA, under the BIC criterion [42]. The best fit model was Hasegawa-Kishino-Yano (HKY), for the ETS multi-alignment, and Tamura 3-parameter (T92), for the *psb*A*-trn*H multi-alignment. Non-parametric bootstrap (BT) re-sampling [43] was performed to test the robustness of the obtained topologies (1000 replicates). Bayesian inference (BI) analyses were also carried out using MrBayes version 3.1.2 [44] with the same best-fitting models employed for ML analyses, after having generated the nexus alignments with Mesquite version 2.71 [45]. The BI analyses consisted in two independent Markov chain Monte Carlo (MCMC) runs, each composed of four chains (three heated and one cold). Each MCMC ran for 5 × 10^6^ generations for the ETS multi-alignment and 1 × 10^6^ generations for the *psb*A*-trn*H multi-alignment, sampling trees every 100 generations. At the end of each run, the posterior distribution was considered adequate if the average standard deviation of the split frequencies was ≤0.01. The first 12,500 trees for the ETS analysis and 2500 trees for the *psb*A*-trn*H analysis were discarded as burn-in, as determined by the stationarity of the lnL using Tracer version 1.5 [46]. The consensus topology and posterior probabilities (PP) were derived from the remaining trees.

For both the ETS and *psb*A*-trn*H datasets, the following species delimitation methods were applied: the Automatic Barcode Gap Discovery (ABGD) method [47], the Assemble Species by Automatic Partitioning (ASAP) method [48], the Poisson Tree Processes (PTP) method [49], and the Generalized Mixed Yule Coalescent (GMYC) method [50]. AGBD was performed on the ETS and *psb*A*-trn*H multi-alignments using the web interface https://bioinfo.mnhn.fr/abi/public/abgd/abgdweb.html (access date: 12 November 2022); Kimura (K80) with TS/TV = 2.0 was selected as distance model and the relative gap width (X) was set at 0.5. ASAP was carried out on the ETS and *psb*A*-trn*H multi-alignments, through the https://bioinfo.mnhn.fr/abi/public/asap/asapweb.html online platform (access date: 14 November 2022); again Kimura (K80) with TS/TV = 2.0 was selected as distance model. PTP was run on the web server http://species.h-its.org/ptp (access date: 15 November 2022), leaving the default values and using the ML phylogenetic tree (without BT statistics) previously obtained for each of the two molecular loci as the input. GMYC was carried out on the http://species.h-its.org/gmyc web interface (access date: 16 November 2022), using an ultrametric tree as the input and selecting the “single threshold” method. For both the ETS and *psb*A*-trn*H multi-alignments, the ultrametric tree was obtained with BEAST version 2.6.3.0 [51], using the same best-fitting models employed for the ML and BI analyses; the “log normal relaxed molecular clock” was selected as clock model, a “coalescence tree with constant population” was chosen as tree model/prior, and the MCMC parameters were the same used for the BI analyses; TreeAnnotator version 1.8.0 [52] and FigTree version 1.4.4 (available at http://tree.bio.ed.ac.uk/software/figtree/) were used to obtain the final ultrametric trees.

Besides the *psb*A*-trn*H dataset, already made up only by the Italian sequences obtained in this study, another ETS dataset was created, including all the sequences obtained in this study plus other published sequences obtained from Italian specimens. These two datasets were used to study and visualize the Italian *Salicornia* haplotypes (also called ribotypes for the ETS locus) with the program PopART version 1.7 [53], which allowed also to plot them on a geographical map. For the haplotype study, the Minimum Spanning Network was selected as network inference method [54].

For the molecular and phylogenetic results, final pictures, suitable for publication, were created with Inkscape version 0.92 (https://www.inkscape.org) and GIMP version 2.8.22 (https://www.gimp.org).

### 2.3. Morphometric Analyses

Four glasswort populations, ascribable to *Salicornia procumbens* according to the molecular analyses and based on Kadereit et al. [7] classification, were subjected to morphometric analyses; each population consisted in 39–41 individuals (Table 1). A total of 28 characters (4 qualitative, 24 quantitative) (Table 2) were measured on 161 individuals (a total of 4508 measurements) using a stereomicroscope Zeiss GXS. The measurements were taken on fresh plant material, since many morphological characters modify after drying. The data matrix (individuals × variables) was processed using the software packages NCSS 2007 and Past 4.11. The variability of the characters has been examined by Principal Component Analysis (PCA), Discriminant Analysis (DA), and Box plots. In PCA analysis, we excluded the qualitative characters. DA was performed using the first five components derived from PCA, which explain about the 72% of the total variability. The use of component scores (each other linearly independent by construction) allows to obtain an unbiased discriminant model, both solving the indeterminacy due to multicollinearity of the independent variables and obtaining a more reliable prediction for the smaller number of involved variables [55,56,57]. As a supervised technique, we performed the DA on groups classified using both the species names and the localities. A multivariate analysis of variance (MANOVA) was also performed to test the significance of differences between responses (dependent) variables (morphological characters) and factor variables (=groups, i.e., taxa). A k-means procedure, which is the most common unsupervised non-hierarchical clustering technique maximizing the between/within-cluster variance ratio (F-Ratio) for a given k number of clusters [58], was performed to identify the optimum number of groups without using any a priori knowledge. A Chi-squared test was performed on a contingency matrix to check for the significance of the correlation of a priori groups and cluster partitions. All the inferential tests were set to *p* < 0.05 as significance threshold.

## 3. Results

### 3.1. Molecular Study

**Figure 2:**
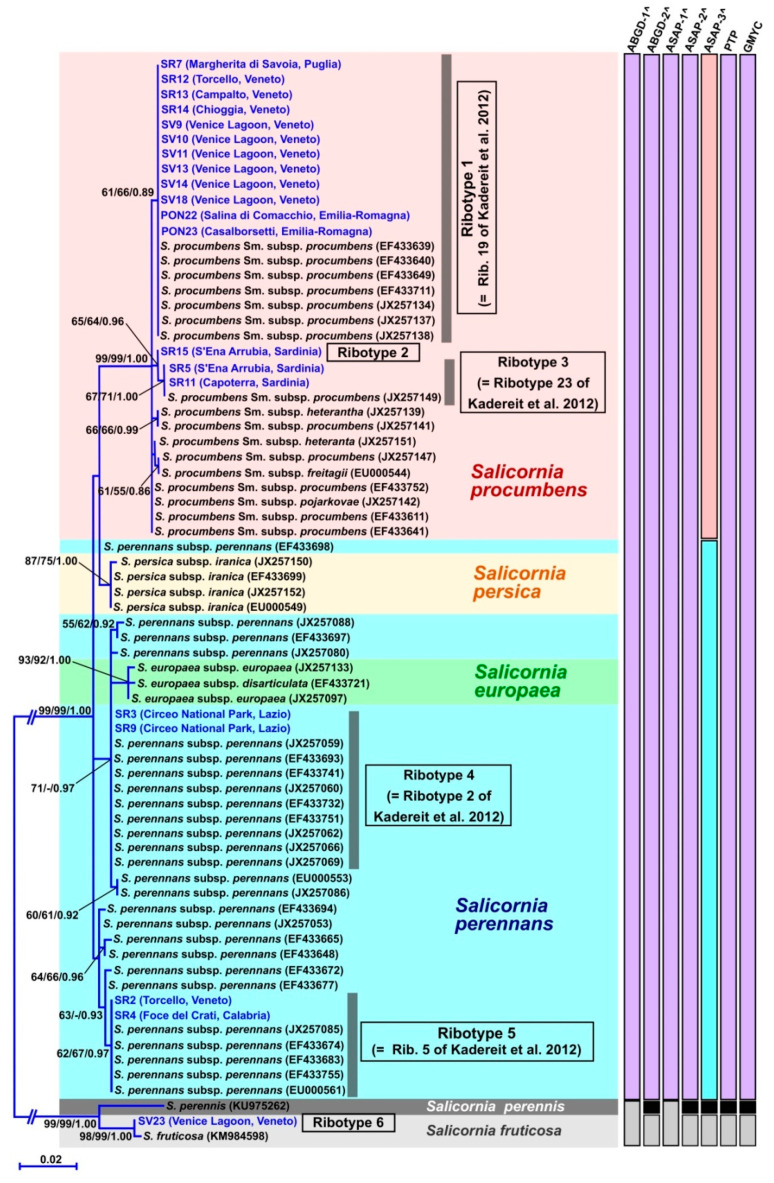
Phylogenetic reconstruction based on the ETS region. For each node, the support values from ML bootstrap and MP bootstrap and BI posterior probabilities are reported, respectively. Only bootstrap supports ≥50% and posterior probabilities ≥0.70 are shown. Values for nodes that obtained support in only one of the phylogenetic analyses were omitted. For each of the downloaded sequences, the species name, followed by the INSDC accession number, is reported. The sequences obtained in this study are highlighted in blue colour and the Italian ribotypes are indicated. Scale bar represents expected number of nucleotide substitutions per site. The results of the species delimitation tests are reported on the right; for ABGD the first two results are reported, and for ASAP the first three results are indicated. Comparison between the detected Italian ribotypes and those of Kadereit et al. [7] were reported.

In the phylogenetic reconstruction based on the nuclear ribosomal locus ETS (Figure 2), the Italian *Salicornia* populations were scattered across three main clades that, based on the most recent classifications, corresponded to the species *Salicornia procumbens*, *Salicornia perennans*, and *Salicornia fruticosa*. More in detail, most of the Italian analyzed samples (almost all those from Veneto, the two from Emilia-Romagna, the one from Puglia and all those from Sardinia) were included in the *S. procumbens* group with other two Italian specimens, whose sequences were published (one from Veneto, Genbank accession: JX257137, and one from Puglia, Genbank accession: EF433640), and several extra-Italian specimens. Inside the *S. procumbens* clade, different lineages were observed, of which three included Italian specimens: (1) a sub-clade corresponding to ribotype 1 (=ribotype 19 of Kadereit et al. [7]), identified by Kadereit et al. [7] as *S. procumbens* subsp. *procumbens*; (2) a lineage represented by the Sardinian specimen SR15 (from S’Ena Arrubia), corresponding to ribotype 2, never reported before; (3) a sub-clade made up of the two Sardinian samples SR5 and SR11 (collected from S’Ena Arrubia and Capoterra, respectively) and a Moroccan specimen (Genbank accession: JX257149), corresponding to ribotype 3 (=ribotype 23 of Kadereit et al. [7]). Four Italian glasswort populations were placed into the *S. perennans* group and divided into two further lineages of this species complex: a sub-group corresponding to ribotype 4 (=ribotype 2 of Kadereit et al. [7]), including specimens SR3 and SR9, both from the Circeo National Park (Lazio), and a sub-clade corresponding to ribotye 5 (=ribotype 5 of Kadereit et al. [7]), including specimen SR2, from Torcello in the Venice Lagoon (Veneto), and specimen SR4, from Foce del Crati (Calabria). Other Italian published sequences were found for the ETS ribotype 5: one from Toscana (Genbank accession: EF433671), two from Puglia (Genbank accessions: EF433673, JX257084), and two from Siciliy (Genbank accessions: EF433670, EF433674; the latter included also in the ETS phylogenetic tree). Finally, specimen SV23 from the Venice Lagoon fell into the clade corresponding to the species *S. fruticosa*.

It is worth to underline that in the ETS phylogenetic reconstruction (Figure 2), while the clades corresponding to the species *S. procumbens* and *S. fruticosa* had high statistical supports (99/99/1.00 and 98/99/1.00, respectively), the clades of other species (e.g., *S. persica* and *S. europaea*) were less supported. Moreover, the specimens attributed to *S. perennans* according to the most recent classifications [7,13] were not included in a supported monophyletic clade, but they divided into more groups with low statistical supports. In addition, the ETS tree region where *S. perennans* specimens were placed showed different unresolved points (Figure 2).

The species delimitation test performed on the ETS sequence dataset (Figure 2) generally evidenced a difficulty to recognize many *Salicornia* species, defined by Kadereit et al. [7] mainly based on morphology and geographical areas. For the ABGD test, two groups of results were reported; they corresponded to partitions with prior values of maximum interspecific divergence (P) ranging from 0.01 to 0.03, since it was demonstrated that partitions with *p* values inside this range generally match real species [47]. In both the ABGD partition types, *S. procumbens*, *S. perennans*, *S. persica*, and *S. europea* were recognized as a unique group; while the two ABGD partitions differed for the fact that, in the first one, *S. perennis* and *S. fruticosa* resulted as a unique clade, and, in the second one, these two lineages were detected as separate groups. For the ASAP test, the three resulting scenarios with lower ASAP scores (the lower the score the more reliable the results [48]) were depicted in Figure 2: the first two ones (with ASAP scores of 1.0 and 3.5, respectively) matched the above described results obtained with the ABGD test, while in the third scenario (ASAP score = 3.5 as well) four Italian *Salicornia* groups were detected: *S. procumbens*; *S. perennans* + *S. persica* + *S. europea*; *S. perennis*; *S. fruticosa* (Figure 2). With both the PTP and one threshold GMYC methods, the unique found partition detected three groups inside the examined Italian glassworts: *S. procumbens* + *S. perennans* + *S. persica* + *S. europea*; *S. perennis*; *S. fruticosa* (Figure 2).

**Figure 3:**
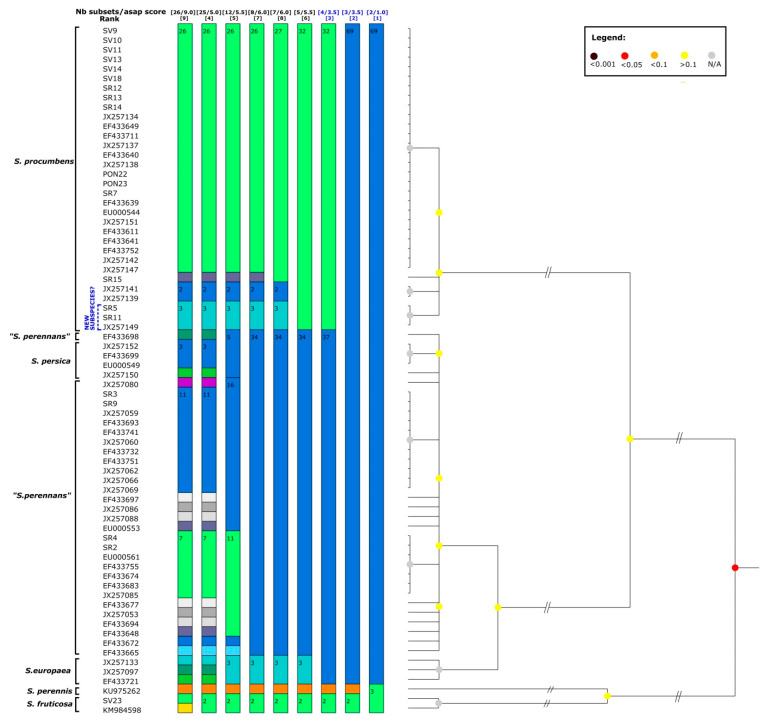
ASAP results for the ETS locus, with the species names reported on the left. In the first line, the blue colour highlights the partitions that are reported on the tree of Figure 2.

In Figure 3, the complete results produced with the ASAP method are reported. It is interestingly to underline that, even if with higher ASAP scores and therefore not depicted in Figure 2, besides the three above-described partitions found with this method, the other six partitions were obtained for a total of nine. In four of these nine partitions, the Sardinian specimen SR15 was highlighted as a separate lineage and, in five of these partitions, the group represented by the Sardinian samples SR5 and SR11 and the Moroccan specimen, already evidenced by the ETS phylogenetic reconstruction, was detected.

**Figure 4:**
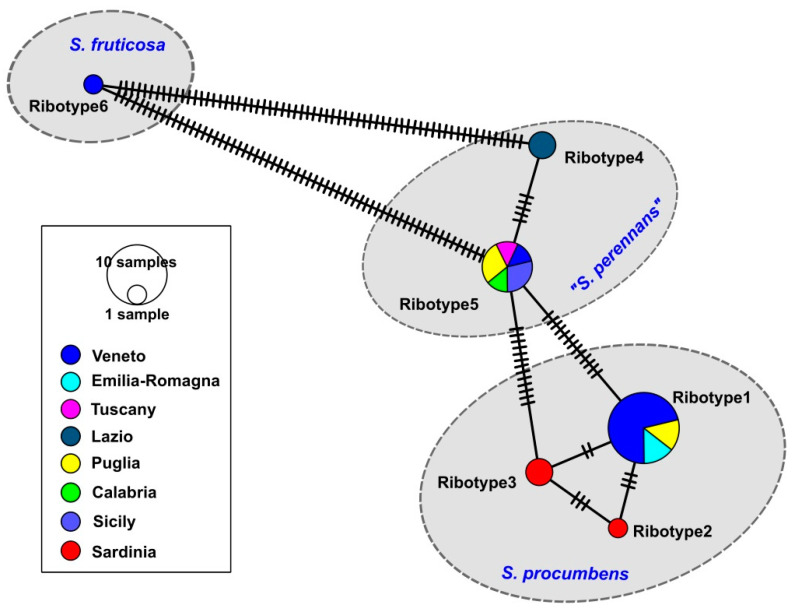
Minimum Spanning Network obtained with the program PopART of the ETS ribotypes found in Italy for the genus *Salicornia*.

**Figure 5:**
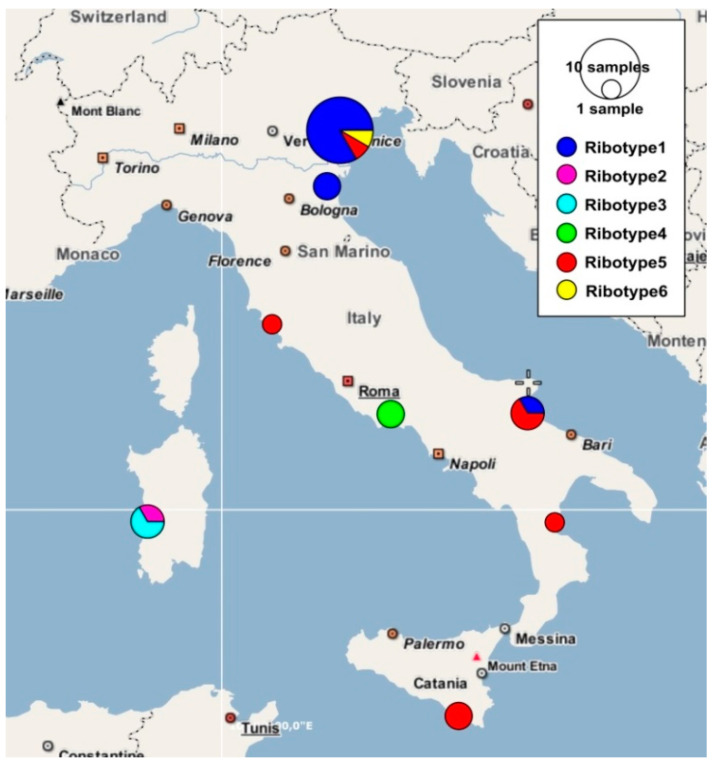
Frequencies of different ETS ribotypes plotted in a geographical map for the genus *Salicornia*.

The ETS ribotype study (Figure 4) showed how the Sardinian specimens (=ribotypes 2 and 3) differed for only few nucleotide substitutions from the other *S. procumbens* specimens collected from Veneto, Emilia-Romagna, and Puglia (=ribotype 1). More in detail, three nucleotide differences distinguished ribotype 1 and ribotype 2, while two nucleotide differences were found between ribotype 1 and ribotype 3. The Sardinian ribotypes 2 and 3 differed for three nucleotides. Many more nucleotide substitutions distinguished the *S. procumbens* ribotypes from the *S. perennans* and *S. fruticosa* ones, with these last the most divergent. Inside the *S. perennans* morphotypes, the ribotypes 4 (from Lazio) and 5 (found in Veneto, Toscana, Puglia, Calabria, and Sicily) differed for five nucleotide substitutions. The ETS ribotype 5 was the most widespread in Italy, being present in five regions, followed by the ETS ribotype 1, present in three regions (Figure 5). Finally, the region showing the highest ETS ribotype diversity was Veneto, where three ribotypes corresponding to three *Salicornia* species (*S. procumbens*, *S. perennans*, and *S. fruticosa*) were detected; however, this could be due to the higher number of samples analyzed for this region.

**Figure 6:**
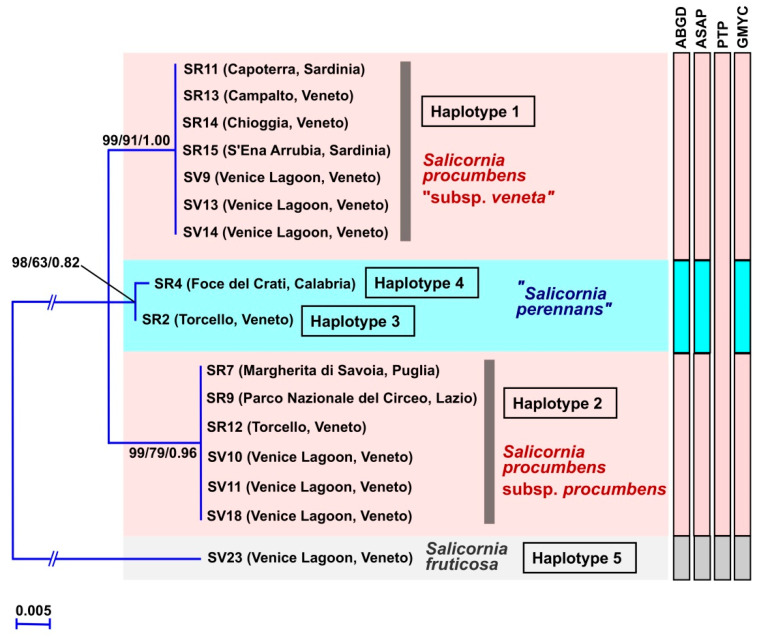
Phylogenetic reconstruction based on the *psb*A*-trn*H intergenic spacer. For each node, the support values from ML bootstrap and MP bootstrap and BI posterior probabilities are reported, respectively. Only bootstrap supports ≥ 50% and posterior probabilities ≥ 0.70 are shown. Values for nodes that obtained support in only one of the phylogenetic analyses were omitted. Scale bar represents expected number of nucleotide substitutions per site. The results of the species delimitation tests are reported on the right.

In the phylogenetic reconstruction based on the plastid *psb*A-*trn*H intergenic spacer (Figure 6), the analyzed Italian glassworts were scattered into four lineages. This result was supported by three (ABGD, ASAP, and GMYC) of the four performed species delimitation tests, with the exception of the PTP test that distinguished just two lineages: the *S. fruticose* lineage and a group composed by all the remaining specimens. Indeed, with the exception of *S. fruticose*, the *psb*A-*trn*H phylogenetic reconstruction did not resolve the evolutionary relationships among the three remaining *Salicornia* lineages (Figure 6). Two of the *psb*A-*trn*H found lineages corresponded to the just cited *S. fruticosa* and to the species *S. perennans*, even if the specimens SR2 and SR4 (=ETS ribotype 5) included in the latter clade exhibited a certain distance between them and, indeed, corresponded to two different plastid *psb*A-*trn*H haplotypes (haplotypes 3 and 4). The remaining Italian *Salicornia* specimens, which were identified as *S. procumbens* in the ETS tree (Figure 2), were divided into two distinct and well-supported clades. The first *S. procumbens* clade (statistical support: 99/91/1.00) included the Venetian specimens SV9, SV13, SV14 (from the Venice Lagoon), SR13 (from Campalto, *locus classicus* of the ex. *S. veneta*), and SR14 (from Chioggia) and the Sardinian specimens SR11 and SR15 (from Capoterra and S’Ena Arrubia, respectively); this clade corresponded to the plastid *psb*A-*trn*H haplotype 1. The second *S. procumbens* clade (statistical support: 99/79/0.96) included the Venetian specimens SV10, SV11, SV18 (from the Venice Lagoon), and SR12 (from Torcello), the Apulian specimen SR7 and, interestingly, the Latial specimen SR9 (from Circeo National Park near Sabaudia), which was ascribable to *S. perennans* on the basis of the ETS phylogenetic reconstructions (Figure 2); this *S. procumbens* clade corresponded to the plastid *psb*A-*trn*H haplotype 2.

**Figure 7:**
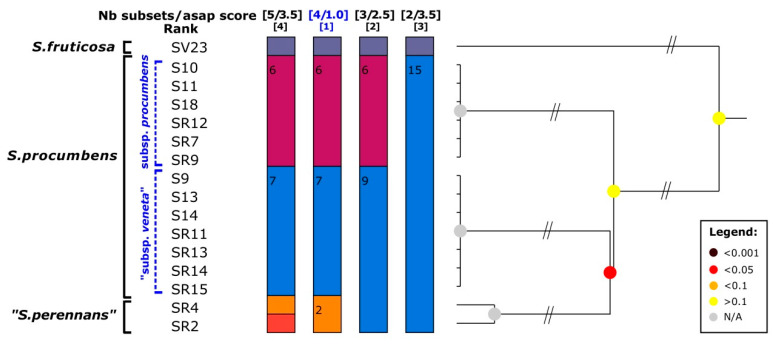
ASAP results for the *psb*A*-trn*H locus, with the species names reported on the left. In the first line, the blue color highlights the partition that is reported on the tree of Figure 6.

In Figure 7 all the partition results found with the ASAP test for the *psb*A-*trn*H dataset are reported, besides the one depicted in Figure 6 that had the lower ASAP score (=1.0). The above-described division of the *S. procumbens* specimens was found also in partition 4 (ASAP score = 3.5), while partition 2 (ASAP score = 2.5) highlighted a less sharp division between “*S. perennas*” and some *S. procumbens* specimens and partition 3 (ASAP score = 3.5) matched the results found with the PTP test.

**Figure 8:**
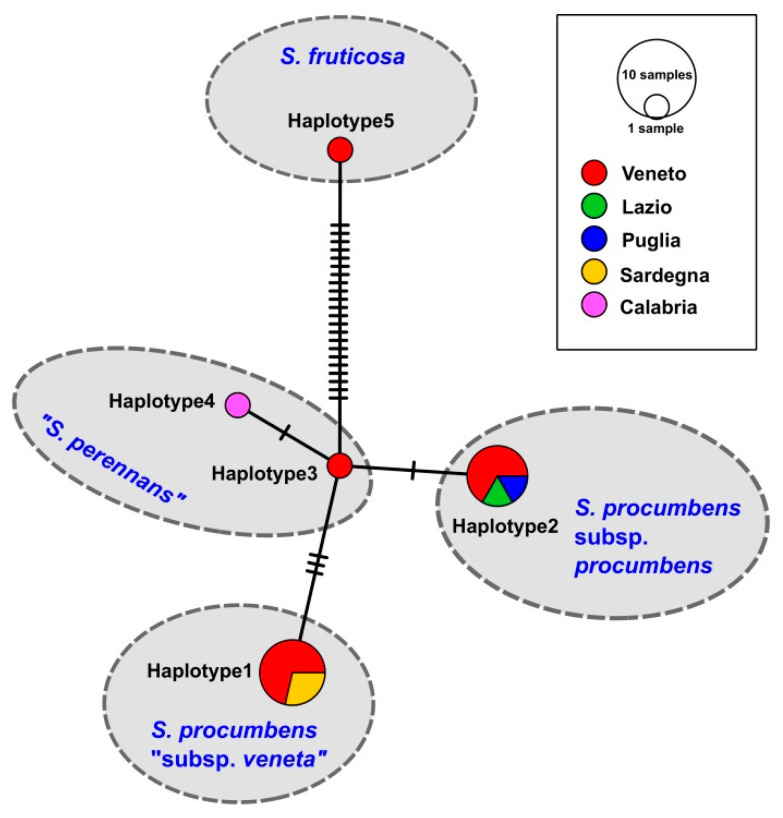
Minimum Spanning Network obtained with the program PopART of the plastid *psb*A*-trn*H haplotypes found in Italy for the genus *Salicornia*.

The *psb*A-*trn*H haplotype study (Figure 8) mirrored the results found with the phylogenetic reconstruction. In particular, the more divergent Italian *Salicornia* plastid haplotype was the one corresponding to the species *S. fruticosa*, which differed for 22–25 nucleotide substitutions from the other haplotypes. The remaining plastid haplotypes evidenced the less sharp division between the assigned species, with the *S. procumbens* haplotype 2 more similar to the “*S. perennans*” haplotypes 3 and 4 (with one and two nucleotide differences, respectively) than to the *S. procumbens* haplotype 1. In fact, the *S. procumbens* haplotype 1 differed for 4 nucleotide substitutions from the *S. procumbens* haplotype 2 and for 3–4 nucleotide substitutions from the “*S. perennans*” haplotypes. Finally, the “*S. perennans*” haplotypes where distinguished for just one nucleotide substitution.

Moreover, all the found plastid *psb*A-*trn*H *Salicornia* haplotypes could be distinguished for the presence of gaps and/or indels, particularly rich in adenine and thymine bases (Supplementary File S1), which could not be considered with the program used for the haplotype study. More in detail, besides the already cited nucleotide differences, *S. fruticosa* haplotypes showed the presence of various long indels respect to the other species haplotypes. The two *S. procumbens* haplotypes were distinguished for five indels: one was one nucleotide long, two were two nucleotides long and two were five nucleotides long. The two “*S. perennans*” haplotypes differed for three indels: two were one nucleotide long and one was seven nucleotides long (Supplementary File S1).

**Figure 9:**
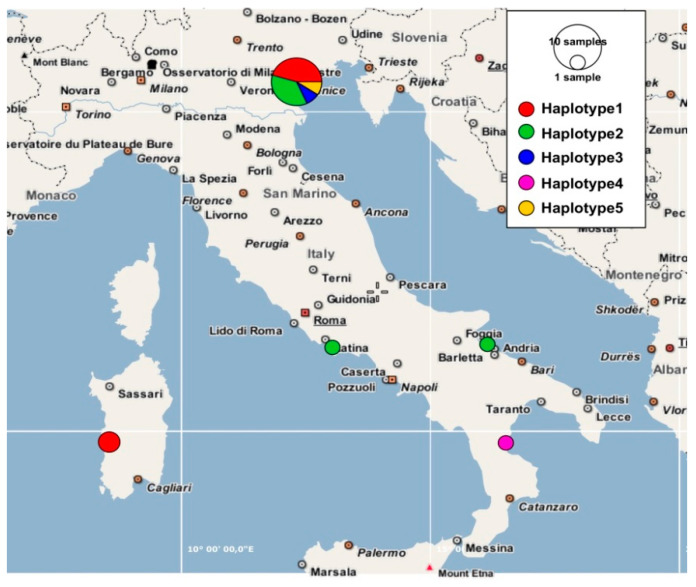
Frequencies of different Italian plastid *psb*A*-trn*H haplotypes plotted in a geographical map for the genus *Salicornia*.

The most widespread Italian plastid haplotype was the *S. procumbens* haplotype 2 (present in three regions), while the region with the highest variety of plastid haplotypes was again Veneto, where four haplotypes were detected (Figure 9).

### 3.2. Morphometric Analyses

**Figure 10:**
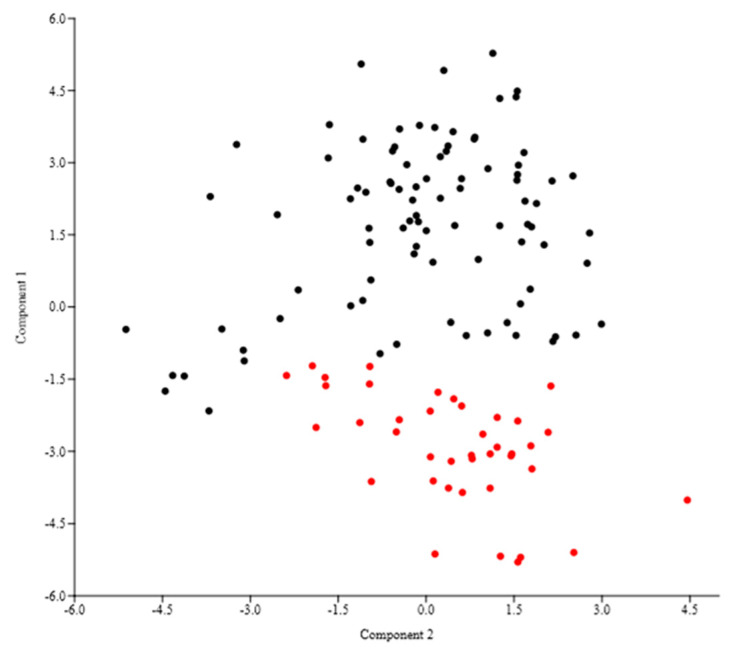
PCA (first (axis x) vs. second (axis y) components) based on the 24 quantitative morphological characters. Black dots: Venetian population; red dots: Sardinian population.

The Principal Component Analyses of the 24 analyzed morphological quantitative characters showed that the cumulative percentage of eigenvalues for the first five axes was 71.46%, with higher contribution (about 62%) given by the first three components (33.20%, 15.79%, and 9.22%, respectively). The examination of the combined graphs among pairs of these five components showed two separated groups along the first component (Figure 10). These groups corresponded to the populations from Veneto region (Campalto (SR13), Chioggia (SR14), and Torcello (SR12) (Table 1)) and Sardinia (S’Ena Arrubia, SR15), respectively. The highest contribution to axes was given by the following characters: height of the side flower of the third segment, length of the spike, distance from the tip of the third fertile segments to apex of middle flower, number of fertile segments, length of the second internode, length of the ultimate internode.

**Figure 11:**
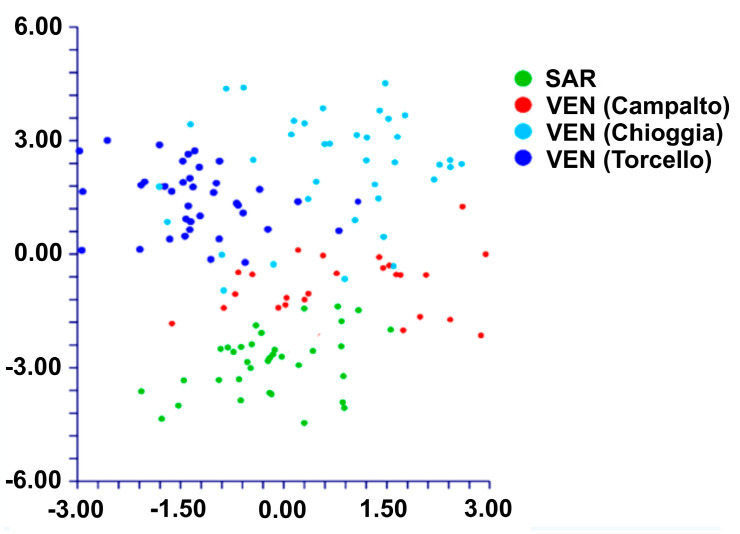
DA (first (axis y) vs. second (axis x) components) performed of groups classified using the localities names.

The DA analysis (Figure 11), carried out using the locality names, predicted two groups based on the first two discriminant functions which explained the 92.5% of the total variation (eigenvalues: 78.4% (1st function) and 14.1% (2nd function)). These groups corresponded to populations from Veneto (localities Campalto, Chioggia, and Torcello, which are more or less overlapped each other) and Sardinia (S’Ena Arrubia), respectively. The value of correct classification was good (75.8%).

The results of MANOVA (Table 3) showed significant differences at both species and populations level. Probability level was less than 0.000001 for all the considered statistics tests (Wilks’ Lambda, Hotelling–Lawley Trace, Pillai’s Trace, and Roy’s Largest Root). F-Ratios were high (18.88).

The K-means clustering procedure (Table 4) suggested a two-clusters solution for the considered individuals. The first component, which gives the higher contribution in PCA analysis (37.00% vs. 16.14% (2nd component), vs. 8.80% (3rd component), vs. 7.28% (4th component), and vs. 5.56% (5th component)), had an F-Ratio of 263.34 in the two-clustered running procedure, whereas F-Ratios were 143.80, 114.46, and 61.76 in the 3-, 4-, and 5-clustered procedures, respectively. Additionally, F-Ratios of the other components increased by imposing 3, 4, or 5 clusters in k-means procedure, thus highlighting a worse grouping if moving from 2 to 3, 4 or 5 clusters (Table 4).

The Chi-squared test values were 50.14 (*p* = 1.43 × 10^−12^), 45.84 (*p* = 1.1 × 10^−10^), 77.52 (*p* = 1.1 × 10^−16^), and 85.99 (*p* < = 1.0 × 10^−16^) for 2, 3, 4, and 5 groups, respectively. These values showed that the most relevant partition suggested by data structure was the separation between Sardinia and Veneto populations almost caught by the two cluster solutions.

**Figure 12:**
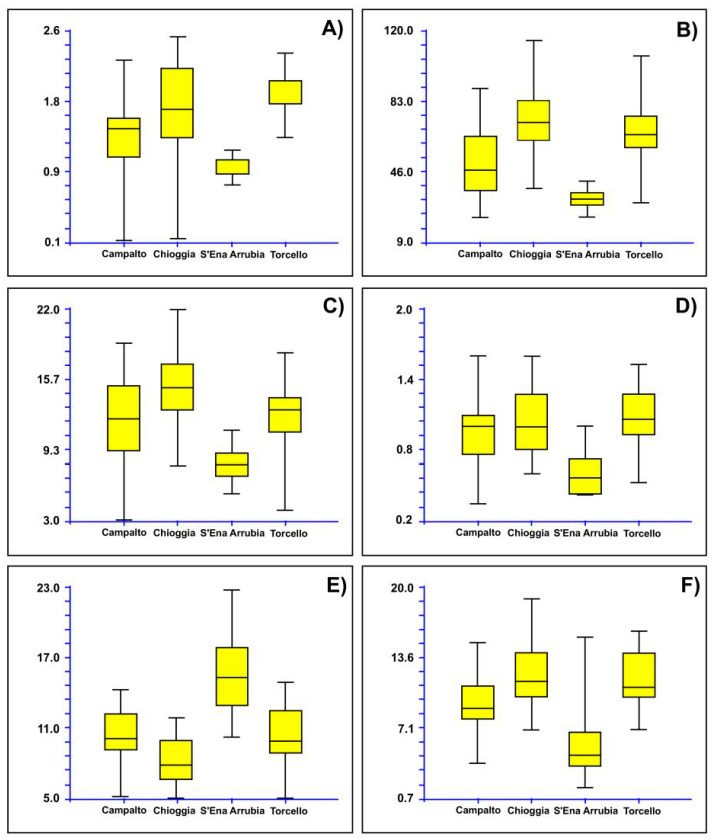
Box plots showing the variability of the diagnostic character derived from PCA: (**A**) height of the side flower of the third segment, (**B**) length of the spike, (**C**) distance from the tip of the third fertile segments to apex of middle flower, (**D**) number of fertile segments, (**E**) length of the second internode, (**F**) length of the ultimate internode. Campalto, Chioggia, and Torcello populations in Venetian lagoon; S’Ena Arrubia population in Sardinia Island.

The box plots (Figure 12), which were performed using the characters derived from PCA analyses that were able to discriminate the various groups (i.e., height of the side flower of the third segment, length of the spike, distance from the tip of the third fertile segments to apex of middle flower, number of fertile segments, length of the second internode, length of the ultimate internode), confirmed the separation of Sardinia population (SR15 in nuclear tree; see Figure 2) from the other ones (Venetian lagoon; i.e., SR12, SR13, and SR14), which appear, in turn, more or less overlapped. As expected, being the specimens deriving by the same taxon, no single character had a relevant discriminant power *per se*, whereas the combination of different features, stemming from their mutual correlations caught by principal component scores, allowed a clear separation between Sardinia and Veneto groups.

Finally, concerning the qualitative characters (color of sterile and fertile segments and of flowers of the third segment and distribution of the coloration along the plant), they did not allow to distinguish any group. Each population displayed all the considered colours.

**Figure 13:**
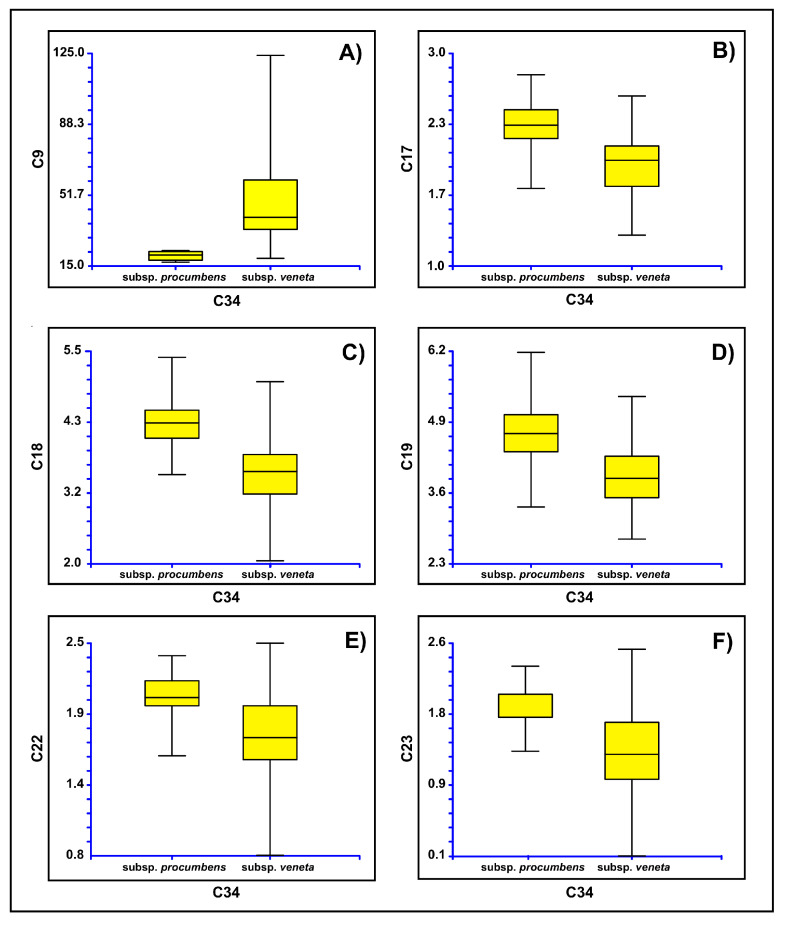
Box plots showing the variability of the diagnostic character for the groups derived from the plastid tree: (**A**) length of the ultimate branch, (**B**) width of the middle flower, (**C**) width of the three flowers, (**D**) minimum width of the second fertile segment, (**E**) height of the middle flower of the third segment, and (**F**) height of the side flower of the third segment.

Box plots were also performed based on of the results derived from the plastid tree (Figure 6), therefore considering two groups. The first one was composed by the populations from Campalto (SR13 according to Figure 6), Chioggia (SR14), and S’Ena Arrubia (SR15), which are attributable to *Salicornia veneta sensu* [15,18]; the second group was the Torcello population (SR12), which would be *S. procumbens* subsp. *procumbens*. Six characters were found to be diagnostic between these two groups: length of the ultimate branch, width of the middle flower, width of the three flowers, minimum width of the second fertile segment, height of the middle flower of the third segment, and height of the side flower of the third segment (Figure 13).

## 4. Discussion

The aim of this study was to investigate different Italian *Salicornia* populations using molecular and morphometric techniques, as up to now most of the studies had been based only on classical approaches and just few published sequences were available for Italian glassworts in the public databases. A particular focus was put on specimens ascribable to *Salicornia procumbens* [10], since in 2012 the species *Salicornia veneta* [16], a taxon endemic to the Italian Adriatic coasts and to western Sardinia [4,14,15,18,19,20,21], was synonymized with *S. procumbens* subsp. *procumbens* [7], even if it is still considered as a distinct species by most Italian authors [15,20,21,29] and in the common practice. Therefore, the debate on the existence of *S. veneta* was still open.

The molecular results obtained with both the considered molecular markers (the nuclear ribosomal ETS and the plastid *psb*A-*trn*H intergenic spacer) detect three main taxonomic entities among the Italian analyzed glassworts, which correspond to three distinct species: *S. procumbens*, *Salicornia perennans* [9], and *Salicornia fruticosa* [13]. With both the molecular loci, *S. fruticosa* results evolutionary the most distant of the detected species, confirming its attribution to a distinct *Salicornia* subgenus (i.e., *Arthrocnemoides* Ung.-Sternb.) proposed by Piiranien et al. [13]. The performed molecular analyses evidence also that *S. perennans* is not monophyletic and that it needs further revision, as already suggested by Kadereit et al. [7]. Therefore, *S. perennans* should currently be considered as a species/subspecies complex, with the borders of the different taxonomic entities belonging to this complex still to be determined. In this regard, it is to underline the ambiguous phylogenetic position of the Latial specimen SR9, which is attributable to *S. perennans*, based on the nuclear ETS locus, and to *S. procumbens*, based on the plastid *psb*A-*trn*H marker. This and other discrepancies between the ETS and *psb*A-*trn*H phylogenetic reconstructions may be due to the fact that the two loci are part of the nuclear and plastid genomes, respectively, and reflect their different evolutionary histories. In fact, it is known that nuclear and plastid genomes can be subjected to several evolutionary processes, among which interspecific hybridization, with the following introgression, and incomplete lineage sorting are frequently found in plant taxa [59,60], further complicating the interpretation of molecular results.

In the *psb*A-*trn*H phylogenetic tree, the Italian glassworts populations attributable to *S. procumbens* with the ETS locus (plus the just cited SR9 specimen) form two well distinct groups, corresponding to the plastid haplotypes 1 and 2. The plastid haplotype 1 is present in different Venetian specimens (including specimen SR13 from Campalto, the *locus classicus* of *S. veneta*) and in all the Sardinian specimens. The plastid haplotype 2 corresponds to the other *S. procumbens* populations collected in Veneto and Puglia and to the specimen SR9 from Lazio. The nucleotide substitution number (i.e., four nucleotide substitutions) found between the plastid haplotypes 1 and 2 of *S. procumbens* is the same or higher than the nucleotide substitution number distinguishing *S. procumbens* haplotype 1 from the “*S. perennans*” haplotypes (i.e., four nucleotide substitutions with “*S. perennans*” haplotype 4 and three nucleotide substitutions with “*S. perennans*” haplotype 3, respectively). Instead, the *S. procumbens* haplotype 2 differs only for 1–2 nucleotides from the “*S. perennans*” haplotypes. The distinction between *S. procumbens* haplotypes 1 and 2 is further evidenced by the indels found between them, which were composed by adenine and thymine bases. It was suggested that the finding of indels, particularly those adenine and thymine rich, in the plastid *psb*A-*trn*H intergenic spacer indicates the presence of different plant species [61]. All these considerations allow us to say that the *psb*A*-trn*H haplotype 1 of *S. procumbens* represents a taxon distinct from the other *S. procumbens* (= plastid haplotype 2) and from all the other Italian glassworts. Moreover, the geographic distribution of the Italian *S. procumbens* with plastid haplotype 1 (i.e., Veneto and Sardinia) induce us to hypothesize that these glasswort populations coincide with the former *S. veneta*. Taken the molecular results as a whole, we suggest recognizing this taxonomic entity at least at the subspecies level, as *S. procumbens* subsp. *veneta* comb. and stat. nov. (see the Taxonomic Treatment below), which in the Veneto region coexists with the populations of *S. procumbens* subsp. *procumbens*.

As hinted above, probably for the different evolution of the nuclear and plastid genomes, in the ETS locus phylogenetic reconstruction, we do not observe the *S. procumbens* division found with the *psb*A-*trn*H marker. Nevertheless, the nuclear locus evidences a further distinction among the Italian *S. procumbens* populations. In fact, the ETS tree shows that the *S. procumbens* from Sardinia are distinct from the other ones analyzed in this study and from the other Italian populations identified by Kadereit et al. [7] as *S. procumbens* subsp. *procumbens*. In particular, two Sardinian ETS ribotypes were found (ribotypes 2 and 3), differing each other for three nucleotide substitutions. Of the Sardinian ribotypes, the ETS ribotype 2 has never been found before, while the ETS ribotype 3 was already reported just for a Moroccan specimen, collected in the Lagoon of Oualida (Western Morocco, Atlantic Ocean), corresponding to ribotype 23 of Kadereit et al. [7]. The ETS lineage represented by ribotype 2 (=SR15) and the sub-clade formed by ribotype 3 specimens (SR5 and SR11 from Sardinia and the Moroccan one) are distinguished also by the ASAP test. In particular, the ETS ribotype 3 sub-clade is detected in five of the nine partitions found by the ASAP test, considered one of the most reliable species delimitation methods [48], even if these partitions have higher ASAP scores. In the ASAP test, the statistically more supported partitions have lower ASAP scores; however, since this method was developed to detect possible species and the Sardinian-Moroccan subclade was found in most of the ASAP scenarios, we suppose that this result can indicate the presence of subspecies or of an incipient speciation process (thus justifying the higher ASAP scores). The fact that, contrary to the ETS locus, the plastid *psb*A-*trn*H marker does not detect this possible new subspecies/incipient speciation stage can be due to the different conservation of nuclear and plastid loci, with the latter usually showing a slower evolution rate [62] and therefore being less suitable to detect more recent events. Indeed, if the hypothesis of the Sardinian subspecies will be confirmed in the future by further data, it would not be the first time that endemic and sub-endemic plant entities of Sardinia, as well as of other Mediterranean islands (such as Corsica and the Balearic archipelago), are found to have originated from or to be linked with extra-Mediterranean areas, such as Africa [63,64].

The morphometric analyses highlight the existence of two Italian *S. procumbens* groups, concerning both the nuclear and the plastid phylogenetic reconstructions. In the former case, the Sardinian population is separated from the Venetian ones; in the latter case, one of the Venetian populations (from Torcello) is distinguished by the remaining ones, represented by Venetian (including the *locus classicus* of *S. veneta*) and Sardinian populations. Six characters were found to be diagnostic between these two groups as shown in Figure 13.

To conclude, the present first extensive study on Italian *Salicornia*, including both molecular and morphometric analyses, has confirmed the presence of some glasswort species classically recognized in Italy and will be the starting point for further similar investigations on Italian glassworts. It has also shed light on the necessity of extending this type of studies particularly in under-investigated regions, where the recognition of plant taxonomic entities is still based only on traditional approaches and/or common practice, without a molecular framework. This can be useful to confirm/reject the presence of historical taxa and to unveil the existence of new morphologically cryptic or semi-cryptic lineages.

## 5. Taxonomic Treatment

***Salicornia procumbens* subsp. *veneta*** (Pignatti and Lausi) K. Sciuto, M.A. Wolf, A. Sfriso, L. Brancaleoni, M. Iberite, and D. Iamonico, *comb. and stat. nov.* ≡ *S. veneta* Pignatti and Lausi in [18]—Holotype: Italy, Barena di Campalto, Laguna di Venezia, 11 November 1964, *Lausi and Pignatti* s.n. (TSB).

Chromosome number: 2*n* = 36.

Morphological characteristics: length of the ultimate branch 36–59 mm, width of the middle flower 1.70–2.15 mm, width of the three flowers 3.25–3.72 mm, minimum width of the second fertile segment 3.35–4.15 mm, height of the middle flower of the third segment 1.57–2.00 mm, and height of the side flower of the third segment 1.00–1.70 mm.

Molecular characteristics: forming a distinct group in the plastid *psb*A-*trn*H intergenic spacer phylogeny (≡ haplotype 1; Figure 6). With a *psb*A-*trn*H intergenic spacer sequence different for 3 to 25 nucleotide substitutions (Figure 8) and various indels from the other Italian *Salicornia psb*A-*trn*H sequences; in particular, distinct for 4 nucleotide substitutions from the *S. procumbens* subsp. *procumbens psb*A-*trn*H sequence.

Molecular vouchers: OM333823 (ETS); OP821357 (*psbA-trnH* intergenic spacer).

Distribution: Endemic to Veneto and Sardinia (Italy); Figure 9.

## Figures and Tables

**Table 1:** Italian *Salicornia* populations considered in the present study. For each sample, the identification code, the sampling site, and the accession numbers of the sequenced molecular markers are reported (a dash indicates that the sequence was not obtained). Asterisks (first column) indicate the populations used for morphometric analyses; RO: Herbarium of the Sapienza University of Rome.

Samples	Locality and Specimens	ETS	*psb*A-*trn*H
SR2	Italy, Veneto region, Venice province, Torcello islet, brackish hollows near the lagoon, 15 October 2005, M. Iberite	OM333820	OP821351
SR3	Italy, Lazio region, Circeo National Park, Sabaudia, 6 October 2005, M. Iberite	OP821341	-
SR4	Italy, Calabria region, Cosenza province, protected area Foce del Crati, 1 November 2004, M. Iberite	OP821342	OP821352
SR5	Italy, Sardinia region, Oristano province, S’Ena Arrubia, edge of the brackish pond, 31 October 2003, M. Iberite	OM333819	-
SR7	Italy, Puglia region, Barletta-Andria-Trani province, Margherita di Savoia, 28 August 2020, M. Iberite	OP821343	OP821353
SR9	Italy, Lazio region, Circeo National Park, Sabaudia, 22 October 1994, M. Iberite	OP821344	OP821354
SR11	Italy, Sardinia region, Cagliari province, Capoterra, 30 October 2003, M. Iberite	OM333821	OP821355
SR12 *	Italy, Veneto region, Venice province, Torcello islet, brackish hollows near the lagoon, 15 October 2005, M. Iberite (RO)	OM333822	OP821356
SR13 *	Italy, Veneto region, Venice province, Campalto, brackish hollows near the lagoon, 15 October 2005, M. Iberite (RO)	OM333823	OP821357
SR14 *	Italy, Veneto region, Venice province, Chioggia, brackish hollows near the lagoon, 16 October 2005, M. Iberite (RO)	OM333824	OP821358
SR15 *	Italy, Sardinia region, Oristano province, S’Ena Arrubia, edge of the brackish pond, 30 September 2012, C. Pepe (RO)	OM333825	OP821359
SV9	Italy, Veneto region, Venice province, North Lagoon, 26 June 2018, A. Sfriso	OP821345	OP821360
SV10	Italy, Veneto region, Venice province, North Lagoon, 26 June 2018, A. Sfriso	OP821346	OP821361
SV11	Italy, Veneto region, Venice province, North Lagoon, 26 June 2018, A. Sfriso	OP821347	OP821362
SV13	Italy, Veneto region, Venice province, North Lagoon, 10 September 2018, A. Sfriso	OP821348	OP821363
SV14	Italy, Veneto region, Venice province, North Lagoon, 10 September 2018, A. Sfriso	OP821349	OP821364
SV18	Italy, Veneto region, Venice province, North Lagoon, 27 September 2018, A. Sfriso	OP821350	OP821365
SV23	Italy, Veneto region, Venice province, North Lagoon, 10 October 2019, A. Sfriso	OM333826	OP821366
PON22	Italy, Emilia-Romagna region, Ferrara province, Salina di Comacchio, 10 August 2022, L. Brancaleoni	OP328927	-
PON23	Italy, Emilia-Romagna region, Ravenna province, Casalborsetti, 10 August 2022, L. Brancaleoni	OP328928	-

**Table 2:** Characters measured for the morphometric analyses. The characters labelled with an asterisk are qualitative, the other ones are quantitative.

**General Morphology**	1. height of the plant (mm)
	2. height of the plant from rooting point to the first branching point (mm)
	3. number of internodes
	4. length of the first internode (mm)
	5. length of the second internode (mm)
	6. length of the penultimate internode (mm)
	7. length of the ultimate internode (mm)
	8. length of the longest first primary branch (mm)
	9. length of the ultimate branch (mm)
	10. maximum number of secondaries on a primary branch
	11. length of longest secondary branch (mm)
	12. length of longest tertiary branch (mm)
**Characters of the Terminal Spike**	13. spike length (mm)
	14. number of fertile segments
	15. number of sterile segments
	16. maximum width of the third fertile segment (mm)
	17. width of the middle flower (the third segment) (mm)
	18. width of the three flowers (the third segment) (mm)
	19. minimum width of the second fertile segment (mm)
	20. maximum width of the second fertile segment (mm)
	21. distance from the tip of the third fertile segments to apex of middle flower (mm)
	22. height of the middle flower of the third segment (mm)
	23. height of the side flower of the third segment (mm)
	24. height of the scarious margin of the second fertile segment (mm)
	25. color of the sterile segments (green or yellow, pinkish, reddish, green with red margin) *
	26. color of the flowers of the third segment (green or yellow, pinkish, reddish, green with red margin) *
	27. color of the fertile segments (green or yellow, pinkish, reddish, green with red margin) *
	28. distribution of coloration (basal or even, apical) *

**Table 3:** MANOVA applied on taxa groups.

Test Statistic	Test Value	F-Ratio	*p* (0.05)
Wilks’ Lambda	0.170952	18.88	0.0000001
Hotelling–Lawley Trace	4.849603	18.88	0.0000001
Pillai’s Trace	0.829048	18.88	0.0000001
Roy’s Largest Root	4.849603	18.88	0.0000001

**Table 4:** K-means procedure performed on 2, 3, 4, and 5 clusters.

No. of Clusters	Component	Between Mean Square	Within Mean Square	F-Ratio
2	1	87.83528	0.3335492	263.34
	2	18.58399	0.9203334	20.19
	3	1.327774	1.081567	1.23
	4	4.31783	1.056084	4.09
	5	1.842877	1.126392	1.64
3	1	45.3238	0.3151879	143.80
	2	13.40127	0.8662724	15.47
	3	30.0381	0.6544046	45.90
	4	8.941694	0.9634211	9.28
	5	2.232657	1.11531	2.00
4	1	31.93618	0.2790256	114.46
	2	30.4127	0.3918748	77.61
	3	23.55327	0.580306	40.59
	4	8.110217	0.9224969	8.79
	5	0.5629421	1.144354	0.49
5	1	21.64586	0.3504851	61.76
	2	25.13895	0.3247632	77.41
	3	17.15698	0.5999466	28.60
	4	14.80934	0.6669765	22.20
	5	12.01765	0.8042226	14.94

## Data Availability

Data sharing is not applicable to this article.

## Supplementary Materials

The following supporting information can be downloaded at: https://www.mdpi.com/article/10.3390/plants12020375/s1. Supplementary File S1: Supplementary file S1-psbA-trnH alignments.
