# Supplementary material for: Molecular and Morphometric Update on Italian Salicornia (Chenopodiaceae), with a Focus on the Species S. procumbens s. l
Source: Plants (Basel). 2023 Jan 13;12(2):375. doi: 10.3390/plants12020375 (PMC9860865; doi:10.3390/plants12020375)
Supplement: Supplementary file 1 [file plants-12-00375-s001.zip › plants-2109264-supplementary.pdf]

**Alignment between all the *psb*A-*trn*H haplotypes**

```
Haplotype5      CTAGACCTAGCGGCTATTGAAGCTCCATCTACAAATGGATAAAGTTTCATTTTTAGTTTA
Haplotype1      CTAGACCTAGCTGCTATTGAAGCTCCATCTACAAATGGATAAAGTTTCATTTTTAGTTTA
Haplotype2      CTAGACCTAGCTGCTATTGAAGCTCCATCTACAAATGGATAAAGTTTCATTTTTAGTTTA
Haplotype3      CTAGACCTAGCTGCTATTGAAGCTCCATCTACAAATGGATAAAGTTTCATTTTTAGTTTA
Haplotype4      CTAGACCTAGCTGCTATTGAAGCTCCATCTACAAATGGATAAAGTTTCATTTTTAGTTTA
                *********** ************************************************

Haplotype5      GTGTATTCGAGTTATTGAATGTAAAGGGGCAGTGCTGGTTTCTTGTTCTGTCAAGAAATT
Haplotype1      GTGTATTCGAATT----AATGTAAAGGGGCAGTGCTGGTTTCTTGTTCTGTCAAGAAATT
Haplotype2      GTGTATTCGAATT----AATGTAAAGGGGCAGTGCTGGTTTCTTGTTCTGTCAAGAAATT
Haplotype3      GTGTATTCGAATT----AATGTAAAGGGGCAGTGCTGGTTTCTTGTTCTGTCAAGAAATT
Haplotype4      GTGTATTCGAATT----AATGTAAAGGGGCAGTGCTGGTTTCTTGTTCTGTCAAGAAATT
                ********** **    *******************************************

Haplotype5      GGTTATTGCTCCTTTACT-----ATTCCAATTTA---TATATATAAGTTTTTAATTTCTT
Haplotype1      GGTTATTGCTCCTTTACTCGTCCAATCCAATTTAATTTATATATAAGTTTTTAATTTATT
Haplotype2      GGTTATTGCTCCTTTACTCGTCCAATCCAATTTAATTTATCTATAAGTTTTTAATTTATT
Haplotype3      GGTTATTGCTCCTTTACTCGTCCAATCCAATTTAATTTATATATAAGTTTTTAATTTATT
Haplotype4      GGTTATTGCTCCTTTACTCGTCCAATCCAATTTAATTTATATATAAGTTTTTAATTTATT
                ******************     * *********   *** **************** **

Haplotype5      TATTCTAATTTATAATGCTTTTTAAAAATTCTAATGCTTTTTTAAATAGGAAAAACTAAG
Haplotype1      T--------------------------ATTCTAATGCTTTTTTAAATAAGAAAAACTAAG
Haplotype2      T--------------------------ATTCTAATGCTTTTTTAAATAAGAAAAACTAAG
Haplotype3      T--------------------------ATTCTAATGCTTTTTTAAATAAGAAAAACTAAG
Haplotype4      T--------------------------ATTCTAATGCTTTTTTAAATAAGAAAAACTAAG
                *                          ********************* ***********

Haplotype5      AATAATGTTTTCTATTTTTATTCTTTTTTATTTGATTTCGGTATGATATTATTCTAGCTT
Haplotype1      AATAATGTTTTCTATTTTTATTCTTTTT-----GATTTCGGTATGATATTATTCTATCTT
Haplotype2      AATAATGTTTTCTATTTTTATTCTTTTT-----GATTTCGGTATGATATTATTCTAGCTT
Haplotype3      AATAATGTTTTCTATTTTTATTCTTTTT-----GATTTCGGTATGATATTATTCTAGCTT
Haplotype4      AATAATGTTTTCTATTTTTATTCTTTTT-----GAATTCGGTATGATATTATTCTAGCTT
                ****************************     ** ******************** ***

Haplotype5      TTTT--------------------------------------------------------
Haplotype1      TTTTGTAATTTTTTT-------TATTAATTTTTAATTATTAAATTATAATTCAAAATTAA
Haplotype2      TTTTGTAATTTTTTT-------------TTATTAATTTAATAATTATAATTCAAAATTAA
Haplotype3      TTTTGTAATTTTTTTTTATTAATTTTTAATTTTAATTATTAAATTATAATTCAAAATTAA
Haplotype4      TTTTGTAATTTTTTT-------TTATTAATTTTAATTATTAAATTATAATTCAAAATTAA
                ****

Haplotype5      ------------TAATTAATTTTT------------------------------------
Haplotype1      TAAACTTTTTTGTAATTAATTTATTATTT-ATTAATTA--ATTTATTAATATTAATTAAA
Haplotype2      TAAACTTTTTTGTAATTAATTTATTAATTAATTTATTAATATTAATTAATATTAATTAAA
Haplotype3      TAAACTTTTTTGTAATTAATTTATTAATT-ATTAATTA--ATTTAT--------------
Haplotype4      TAAACTTTTTTGTAATTAATTTATTAATT-ATTAATTA--ATTTAT--------------
                            ********** **

Haplotype5      ------TATTAAGAATTAATAAAAGATTTATTAAAGATTTAATTTGTATAATAATTTAAT
Haplotype1      GAAAGATAATATTAATTAAAGAAAGATCGATTAAAGATTAAGTTAGTATAATAATTACAT
Haplotype2      GAAAGATAATATTAATTAAAGAAAGATCGATTAAAGATTAAATTAGTATAATAATTACAT
Haplotype3      ------TAATATTAATTAAAGAAAGATCGATTAAAGATTAAATTAGTATAATAATTACAT
Haplotype4      ------TAATATTAATTAAAGAAAGATCGATTAAAGATTAAATTAGTATAATAATTACAT
                      ** **  ******  ******  ********** * ** ***********  **

Haplotype5      TATTATACAGTTCATTTTTTTGTAAGTAAATTCCGATAAGAAAGGGGCGGATG-TAGCCA
Haplotype1      TATTATACAGTGAATTTGTTTGTAAGGAAATTCAGATAAGAAAGGGGCGGATG-TAGCCA
Haplotype2      TATTATACAGTGAATTTGTTTGTAAGGAAATTCAGATAAGAAAGGGGCGGATG-TAGCCA
Haplotype3      TATTATACAGTGAATTTG-TTGTAAGGAAATTCAGATAAGAAAGGGGCGGATGTTAGCCA
```

```
Haplotype4      TATTATACAGTGAATTTGTTTGTAAGGAAATTCAGATAAGAAAGGGGCGGATG-TAGCCA
                ***********  ****  ******* ****** ******************* ******

Haplotype5      AGTGGATCAAGGCA
Haplotype1      AGTGGATCAAGGCA
Haplotype2      AGTGGATCAAGGCA
Haplotype3      AGTGGATCAAGGCA
Haplotype4      AGTGGATCAAGGCA
                **************
```

**Alignment between *psb*A-*trn*H haplotypes 1 and 2**

```
Haplotype1      CTAGACCTAGCTGCTATTGAAGCTCCATCTACAAATGGATAAAGTTTCATTTTTAGTTTA
Haplotype2      CTAGACCTAGCTGCTATTGAAGCTCCATCTACAAATGGATAAAGTTTCATTTTTAGTTTA
                ************************************************************

Haplotype1      GTGTATTCGAATTAATGTAAAGGGGCAGTGCTGGTTTCTTGTTCTGTCAAGAAATTGGTT
Haplotype2      GTGTATTCGAATTAATGTAAAGGGGCAGTGCTGGTTTCTTGTTCTGTCAAGAAATTGGTT
                ************************************************************

Haplotype1      ATTGCTCCTTTACTCGTCCAATCCAATTTAATTTATATATAAGTTTTTAATTTATTTATT
Haplotype2      ATTGCTCCTTTACTCGTCCAATCCAATTTAATTTATCTATAAGTTTTTAATTTATTTATT
                ************************************ ***********************

Haplotype1      CTAATGCTTTTTTAAATAAGAAAAACTAAGAATAATGTTTTCTATTTTTATTCTTTTTGA
Haplotype2      CTAATGCTTTTTTAAATAAGAAAAACTAAGAATAATGTTTTCTATTTTTATTCTTTTTGA
                ************************************************************

Haplotype1      TTTCGGTATGATATTATTCTATCTTTTTTGTAA-TTTTTTTTATTAATTTTTAATTATTA
Haplotype2      TTTCGGTATGATATTATTCTAGCTTTTTTGTAATTTTTTTTTATTAA--TTTAAT-----
                ********************* *********** *************  ******

Haplotype1      AATTATAATTCAAAATTAATAAACTTTTTTGTAATTAATTTATT-----ATTTATTAATT
Haplotype2      AATTATAATTCAAAATTAATAAACTTTTTTGTAATTAATTTATTAATTAATTTATTAAT-
                ********************************************     **********

Haplotype1      AATTTATTAATATTAATTAAAGAAAGATAATATTAATTAAAGAAAGATCGATTAAAGATT
Haplotype2      -ATTAATTAATATTAATTAAAGAAAGATAATATTAATTAAAGAAAGATCGATTAAAGATT
                 *** *******************************************************

Haplotype1      AAGTTAGTATAATAATTACATTATTATACAGTGAATTTGTTTGTAAGGAAATTCAGATAA
Haplotype2      AAATTAGTATAATAATTACATTATTATACAGTGAATTTGTTTGTAAGGAAATTCAGATAA
                ** *********************************************************

Haplotype1      GAAAGGGGCGGATGTAGCCAAGTGGATCAAGGCA
Haplotype2      GAAAGGGGCGGATGTAGCCAAGTGGATCAAGGCA
                **********************************
```

**Alignment between *psb*A-*trn*H haplotypes 3 (=SR2) and 4 (=SR4)**

```
SR4             CTAGACCTAGCTGCTATTGAAGCTCCATCTACAAATGGATAAAGTTTCATTTTTAGTTTA
SR2             CTAGACCTAGCTGCTATTGAAGCTCCATCTACAAATGGATAAAGTTTCATTTTTAGTTTA
                ************************************************************

SR4             GTGTATTCGAATTAATGTAAAGGGGCAGTGCTGGTTTCTTGTTCTGTCAAGAAATTGGTT
SR2             GTGTATTCGAATTAATGTAAAGGGGCAGTGCTGGTTTCTTGTTCTGTCAAGAAATTGGTT
                ************************************************************

SR4             ATTGCTCCTTTACTCGTCCAATCCAATTTAATTTATATATAAGTTTTTAATTTATTTATT
SR2             ATTGCTCCTTTACTCGTCCAATCCAATTTAATTTATATATAAGTTTTTAATTTATTTATT
```

```
                    ************************************************************

SR4                 CTAATGCTTTTTTAAATAAGAAAAACTAAGAATAATGTTTTCTATTTTTATTCTTTTTGA
SR2                 CTAATGCTTTTTTAAATAAGAAAAACTAAGAATAATGTTTTCTATTTTTATTCTTTTTGA
                    ************************************************************

SR4                 ATTCGGTATGATATTATTCTAGCTTTTTTGTAATTTTTTTTTA-------TTAATTTTAA
SR2                 TTTCGGTATGATATTATTCTAGCTTTTTTGTAATTTTTTTTTATTAATTTTTAATTTTAA
                     ******************************************       **********

SR4                 TTATTAAATTATAATTCAAAATTAATAAACTTTTTTGTAATTAATTTATTAATTATTAAT
SR2                 TTATTAAATTATAATTCAAAATTAATAAACTTTTTTGTAATTAATTTATTAATTATTAAT
                    ************************************************************

SR4                 TAATTTATTAATATTAATTAAAGAAAGATCGATTAAAGATTAAATTAGTATAATAATTAC
SR2                 TAATTTATTAATATTAATTAAAGAAAGATCGATTAAAGATTAAATTAGTATAATAATTAC
                    ************************************************************

SR4                 ATTATTATACAGTGAATTTGTTTGTAAGGAAATTCAGATAAGAAAGGGGCGGATG-TAGC
SR2                 ATTATTATACAGTGAATTTG-TTGTAAGGAAATTCAGATAAGAAAGGGGCGGATGTTAGC
                    ******************** ********************************** ****

SR4                 CAAGTGGATCAAGGCA
SR2                 CAAGTGGATCAAGGCA
                    ****************
```
